# Effects of new polymorphisms in the bovine myocyte enhancer factor 2D (*MEF2D*) gene on the expression rates of the *longissimus dorsi* muscle

**DOI:** 10.1007/s11033-012-1689-6

**Published:** 2012-06-20

**Authors:** E. Juszczuk-Kubiak, R. R. Starzyński, T. Sakowski, K. Wicińska, K. Flisikowski

**Affiliations:** 1 Department of Molecular Cytogenetics, Polish Academy of Sciences Institute of Genetics and Animal Breeding, Jastrzębiec, 05-552 Magdalenka, Poland; 2 Department of Molecular Biology, Polish Academy of Sciences Institute of Genetics and Animal Breeding, Jastrzębiec, 05-552 Magdalenka, Poland; 3 Chair of Livestock Biotechnology, Technische Universität München, Liesel-Beckmannstr 1, 85354 Fresing, Germany; 4 Department of Animal Sciences, Polish Academy of Sciences Institute of Genetics and Animal Breeding, Jastrzębiec, 05-552 Magdalenka, Poland

**Keywords:** *MEF2D*, Polymorphism, Promoter region, Western blot, Real-time PCR, Cattle

## Abstract

Myocyte enhancer factor 2D (MEF2D), a product of the *MEF2D* gene, belongs to the myocyte enhancer factor 2 (MEF2) protein family which is involved in vertebrate skeletal muscle development and differentiation during myogenesis. The aim of the present study was to search for polymorphisms in the bovine *MEF2D* gene and to analyze their effect on *MEF2D* mRNA and on protein expression levels in the *longissimus dorsi* muscle of Polish Holstein–Friesian cattle. Overall, three novel variations, namely, insertion/deletion g.−818_−814AGCCG and g.−211C<A transversion in the promoter region as well as g.7C<T transition in the 5′untranslated region (5′UTR), were identified by DNA sequencing. A total, 375 unrelated bulls belonging to six different cattle breeds were genotyped, and three combined genotypes (Ins-C-C/Ins-C-C, Del-A-T/Del-A-T and Ins-C-C/Del-A-T) were determined. The frequency of the combined genotype Ins-C-C/Ins-C-C and Del-A-T/Del-A-T was varied between the breeds and the average frequency was 0.521 and 0.037, respectively. Expression analysis showed that the *MEF2D* variants were highly correlated with *MEF2D* mRNA and protein levels in the *longissimus dorsi* muscle of Polish Holstein–Friesian bulls carrying the three different combined genotypes. The highest *MEF2D* mRNA and protein levels were estimated in the muscle of bulls with the Ins-C-C/Ins-C-C homozygous genotype as compared to the Del-A-T/Del-A-T homozygotes (*P* < *0.01*) and Ins-C-C/Del-A-T heterozygotes (*P* < *0.05*). A preliminary association study showed no significant differences in the carcass quality traits between bulls with various *MEF2D* combined genotypes in the investigated population of Polish Holstein–Friesian cattle.

**Electronic supplementary material:**

The online version of this article (doi:10.1007/s11033-012-1689-6) contains supplementary material, which is available to authorized users.

## Introduction

The myocyte enhancer factor 2 (*MEF2*) transcription factors family has been shown to play a crucial role in the activation of muscle-specific gene transcription in skeletal, cardiac, and smooth muscle cells [1]. The products of four *MEF2* genes—*MEF2A*, *MEF2B*, *MEF2C* and *MEF2D* are bound as homo- and heterodimers to an A/T-rich DNA consensus sequences and are associated with many muscle-specific genes in vertebrates, such as α-actin, α-myosin heavy chain, cardiac troponins T, C and I, dystrophin, desmin or Ca^2+^ -ATPase [2]. In addition, *MEF2* factors are involved in the regulation of inducible gene expression during myocardial cell hypertrophy, e.g. they are required for MLC2 expression during PE-mediated and ET-1-mediated hypertrophy [3]. Furthermore, *MEF2* factors are indispensable for the development and function of the nervous system, because they regulate neuronal proliferation, differentiation, survival, and synapse development [4]. During myogenesis in skeletal muscle cells, *MEF2C* is expressed within the somite myotome beginning at about 9 days postcoitus (d.p.c.) and *MEF2A* and *MEF2D* are expressed immediately after [5]. The *MEF2* transcription factors play a central role in the control of skeletal muscle development by enhancing the muscle inducing activity of myogenic bHLH proteins. Promoters of the *myogenin* and *Mrf4* genes contain MEF2 binding sites that provide a mechanism for amplifying and maintaining expression and stabilizing the muscle phenotype [1]. Several reports showed that *MEF2* genes and calcineurin may be responsible for the formation of slow-twitch fibers [6, 7], thus suggesting their important role in regulating muscle fiber type composition. Recently, Zhao et al. [8] confirmed that *MRF* and *MEF2* families are crucial for the phenotypic differences between two pig breeds and proposed a novel model of myogenesis. According to these authors, *MyoD* and *MEF2A* control the balance between intermuscular adiopogenesis and myogenesis by regulating CCAAT/enhancer-binding protein (C/EBP) family, while *MEF2C* and myogenic factor 5 (*Myf5*) are important during the whole myogenesis process and *MEF2D* affects muscle growth and maturation.

The bovine *MEF2D* gene has been mapped to chromosome 3 (BTA3) within the QTL region for several meat and carcass quality traits (e.g. backfat thickness, intramuscular fat, body weight and carcass weight) and might be considered as a positional candidate for carcass and meat quality traits in cattle [9, 10]. Their roles in muscle growth and development make *MEF2* genes potential candidates for molecular markers of meat production and carcass quality traits in livestock. However the polymorphism of the *MEF2* genes and its potential effect on gene expression level and muscle growth and development has not yet been thoroughly studied.

Thus, the objective of this study was to identify polymorphisms in the promoter region and 5′UTR of the bovine *MEF2D* gene and investigate their possible effect on the *MEF2D* mRNA and protein levels in the *longissimus dorsi* muscle. Moreover, preliminary association analysis between the polymorphisms and carcass quality traits of Polish Holstein–Friesian bulls was performed.

## Materials and methods

### Animals, tissue and blood sampling, RNA and DNA isolation, cDNA preparation

A group of 203 Polish Holstein–Friesian bulls, a progeny of 24 AI sires, was used to investigate the association between *MEF2D* gene polymorphism and carcass quality traits. Animals were housed in a tie-stall and fed with silage, hay and concentrate ad libitum with constant access to water. After 24 h fattening bulls were slaughtered at the age of 12 months and a body weight of about 370 kg. After cooling for 24 h, the weights of both carcass sides were recorded and the right sides were separated into lean meat, bones and fat, as described previously [11]. The carcass quality traits data included weight of lean in valuable cuts (WLVC), weight of fat in valuable cuts (WFVC), percent of lean in valuable cuts (PLVC) and percent of fat in valuable cuts (PFVC).

Samples of *longissimus dorsi* muscle for qPCR (8 samples from each genotype) and western blot analyses (3 samples from each genotype) were harvested and snap-frozen in liquid nitrogen and stored at −80 °C. Total RNA was extracted from tissues using a Qiagen RNeasy^®^ Fibrous Tissue Mini Kit (Qiagen), according to the manufacturer’s instructions. The quality and quantity of RNA was verified using NanoDrop spectrophotometer (Wilmington, DE) and gel electrophoresis. Reverse transcription was performed on 1 μg of total RNA using Transcription First Strand cDNA Synthesis Kit with oligo(dT) primers (Roche), according to the manufacturer’s protocol. cDNA was stored at −20 °C until use. To investigate the genotype and allelic frequencies, blood samples were collected from 375 unrelated bulls of different breeds: Charolaise (CH; *n* = 35), Hereford (HH; *n* = 34), Limousine (LM; *n* = 27), Simmental (SM; *n* = 29), Polish Holstein–Friesian (HO; *n* = 203) and Polish Red (RP; *n* = 47). Genomic DNA was subsequently extracted from blood samples using Wizard® Genomic DNA Purification Kit (Promega) and stored at −20 °C. All procedures carried out on animals were approved by the Local Ethics Commission, permission No. 29/2007.

### Genomic variants detection and polymorphism analyses

Basing on the genomic sequence of the bovine chromosome 3 (NW_003103861) and human sequence of the chromosome 1 (AL365181.24) using the ScanGen (http://genes.mit.edu/GENSCAN.html) and Apollo sequence annotation editor (http://apollo.berkeleybop.org/current/install.html) six overlapping DNA fragments were designed to amplify the promoter region and the 5′UTR of the *MEF2D* gene (Table S1). Polymerase chain reactions (PCRs) were performed according to standard manufacturer’s protocol (Qiagen). The polymorphism screening was performed using a comparative resequencing approach in 20 bulls representing Polish Holstein–Friesian, Limousine, Hereford and Polish Red breeds. PCR products were sequenced using a 3130xl Genetic Analyzer (Applied Biosystems Applera). The *MEF2D* genotyping was conducted with the use of multitemperature single strand chain polymorphism (MSSCP) method. MSSCP electrophoresis was carried out in Pointer System (Kucharczyk Co.,) at constant power (40 W) for 70 min. Electrophoresis temperatures were as follows: 35, 15 and 5 °C for 350Vh. Gels were subsequently silver stained for 30 min using the Silver Stain Kit (Kucharczyk Co.,) and scanned with Molecular Imager System FX (BioRad). The polymorphism sites were analyzed by sequence comparisons using Clustal W (http://www.ebi.ac.uk/tools/msa/clustalW2) and Chromas Lite v2.01 programs (http://www.technelysium,com.au/chromas). Genotype and haplotype frequencies and deviation from the Hardy–Weinberg equilibrium were calculated using POPGENE V3.1 software (http://www.ualberta.ca/~fyech). Searching for putative binding sites for transcription factors was carried out using TESS software (http://www.cbil.upenn.edu/cgi-bin/tess/tess).

### Real-time PCR

qPCR amplification was done in triplicate, using a SYBR Green detection and the Roche Light Cycler 2.0 system (Roche). Real-time PCR primers were designed to anneal to adjacent exons or exon–exon junctions (Table S1). Raw results were normalised relative to the geometric mean of mRNA detected from three reference genes *SF3AI*, *EEFIA2* and *TBP* genes. The gene relative expression levels were evaluated with the use of comparative *Ct* (∆∆*Ct*) value method [12]. The ∆*Ct* values were calculated by subtracting the geometric mean *Ct* value of three reference genes from the target *Ct* value for each sample. The significance of the differences between the expression levels of the *MEF2D* genotypes was estimated using Duncan’s test.

### Western blot analysis

For the detection of MEF2D protein, nuclear extracts were prepared from frozen *longissimus dorsi* muscle, according to Andrews and Faller [13]. Nuclear extracts (80 μg) were subsequently resolved on 10 % SDS–polyacrylamide gel and transferred to PVDF Immobilon-P Transfer Membrane (Millipore). The membranes were initially blocked by gentle agitation in TBST (0.15 % Tween 20 in Tris-buffered saline) containing 5 % fat-free dried milk for 1 h at room temperature followed by overnight incubation at 4 °C with the mouse monoclonal antibody specific for bovine MEF2D (*sc*-*136196*; Santa Cruz Biotechnology). Membranes were then washed and incubated with peroxidase-conjugated anti-mouse antibody (Santa Cruz Biotechnology) for 1 h at room temperature. Immunoreactive bands were detected using the Immobilon™ Western Chemiluminescent HRP Substrate (Millipore) according to the manufacturer’s instructions. Quantification using BioRad Molecular Imager FX based on Quantity One software (BioRad) was performed relative to β-*actin* detected using a specific antibody (Santa Cruz Biotechnology). Reactions were carried out in triplicate for each sample. The differences were tested using Duncan’s test.

### Association studies

The association between genotypes of the *MEF2D* gene and carcass quality traits was analyzed by the least-squares method as applied in the general linear model (GLM) procedure of SAS (SAS, 2004) according to the model: *Y*_*ijkl*_ = *μ* + *G*_*i*_ + *SY*_*j*_ + *S*_*k* +_ *β*(*x*_*ijkl*_−*x*) + *e*_*ijkl*_, where: *Y*_*ijkl*_—studied traits; μ—overall mean; *G*_*i*_—the fixed effect of *i*-th genotype of the *MEF2D* gene (*j* = 1,., 3); *SY*_*j*_—the fixed effect of *j*-th year and season at start of fattening (*k* = 1,., 3); *S*_*k*_—the random effect of *k*-th sire; *β*(*x*_*ijkl*_ *−x*)—the regression of the analyzed trait on the cold carcass weight; *e*_*ijkl*_—the random residual effect. Significant differences in carcass trait levels between bulls with different genotypes were verified with the Duncan’s test.

## Results

**Fig. 1:**
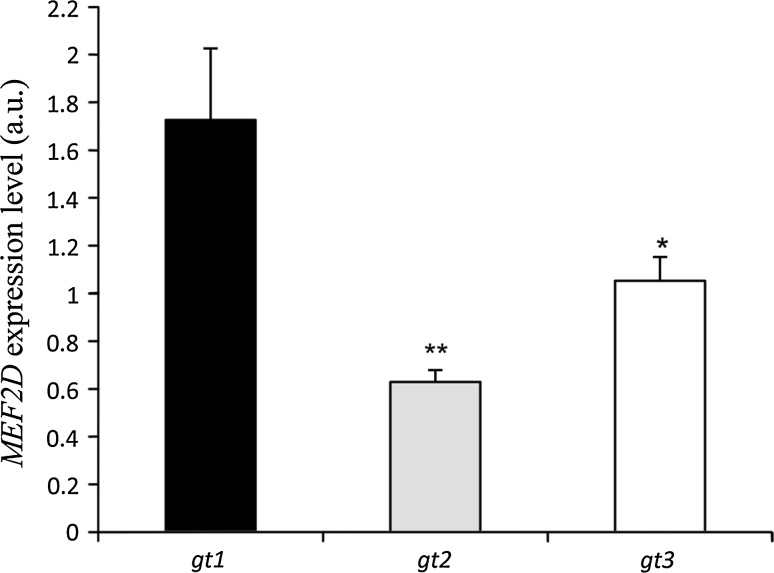
qPCR analysis showing the effect of −818_−814AGCCGIns/Del, g.−212C<A and g.7C<T polymorphisms of the *MEF2D* gene on its mRNA level in the *longissimus dorsi* muscle of Polish Holstein–Friesian bulls. Combined genotypes: *gt1*—(Ins-C-C/Ins-C-C), *gt2*—(Del-A-T/Del-A-T), *gt3*—(Ins-C-C/Del-A-T). Eight samples for each genotype were analysed; *P ≤ *0.05*, **P < *0.01*

**Fig. 2:**
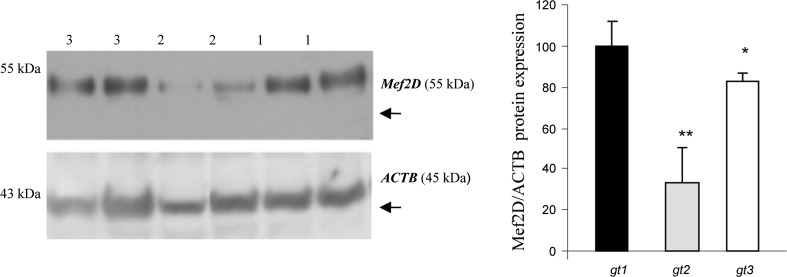
Western blot analysis of the MEF2D protein level in the *longissimus dorsi* muscle of bulls with different *MEF2D* combined genotypes: *gt1*—(Ins-C-C/Ins-C-C), *gt2*—(Del-A-T/Del-A-T), *gt3*—(Ins-C-C/Del-A-T). Three samples for each genotype were analysed; *P ≤ *0.05*, **P < *0.01*

A total of 1791 bp, encompassing the promoter and 5′untranslated region (5′UTR) of the bovine *MEF2D* gene, were resequenced, thus resulting in the detection of three novel variants, more specifically g.−818_−814AGCCG Ins/Del and g.−211C<A SNP in the promoter region as well as g.7C<T SNP in the 5′UTR (Fig. S1). The nucleotide sequences with polymorphic sites have been deposited in the GenBank database under accession no. JQ901405 and JQ901404. By applying the MSSCP method, 375 unrelated bulls representing six cattle breeds (HO, RP, HH, CH, LM and SM) were genotyped, and three genotypes for each locus were identified. At the g.−818_−814 locus, AGCCG insertion was predominant in all of the examined breeds of cattle, except for the LM cattle, for which a lower frequency of allele C at g.−211C<A and g.7C<T loci, respectively, was also noted. Frequency of genotypes was varied between the tested breeds, thus indicating a higher frequency of homozygotes Ins/Ins at position −818_−814, CC at position −212 and CC at position 7 in the HO, PR, and CH breeds. However, heterozygotes Ins/Del, CA and CT for these loci occurred more frequently in the HH, SM and LM breeds. A low frequency of the Del/Del, AA and TT genotypes at each of these loci was observed in the HO, HH, LM and CH breeds and these genotypes were not detected in the RP and SM breeds. The genotype and allele frequencies for individual variations in each breed are summarized in Table 1. Genotype distributions did not deviate from the Hardy–Weinberg equilibrium, with the exception of the RP breed (*P* < *0.05*). The distribution of genotypes in individual animals revealed that homozygotes with insertion AGCCG at position −818_814 were homozygous CC at position −121 and 7, whereas homozygotes with a deletion of AGCCG at position −739_734 were homozygous AA at position −121 and TT at position 7, respectively. Based on these results, three combined genotypes, Ins-C-C/Ins-C-C, Del-A-T/Del-A-T and Ins-C-C/Del-A-T, were determined with a higher frequency of genotype In-C-C/In-C-C in the HO (58.6 %), RP (55.3 %) and CH (62.9 %). The Ins-C-C/Del-A-T genotype was predominant in the HH (50.0 %), SM (51.9 %) and LM (55.6 %) breeds (Table 2). Frequency of the Del-A-T/Del-A-T genotype was low in all of the examined breeds of cattle. In silico analysis of the promoter SNPs using TESS software revealed that the A allele at g.−211C<A SNP lacked putative binding sites for *Sp1*, *AP2*, *AP*-*alpha*, *AP*-*2alphaB* transcription factors, while deletion AGCCG at position −818_−814 disrupted the putative binding site for the *RAF* transcription factor (Fig. S2). In silico transcription factor binding site analysis was in line with qPCR and the western blot results, which showed genotype-dependent *MEF2D* mRNA and protein levels in the *longissimus dorsi* muscle of Polish Holstein–Friesian bulls. Lower *MEF2D* mRNA (Fig. 1) and MEF2D protein (Fig. 2) levels were detected in the muscle tissue of animals carrying the homozygous Del-A-T/Del-A-T genotype than in those with the homozygous Ins-C-C/Ins-C-C (*P* < *0.01*) and heterozygous Ins-C-C/Del-A-T (*P* < *0.05*) variants. Preliminary association analysis showed that the *MEF2D* variants had no statistically significant effect on the carcass quality traits of 203 bulls belonging to the Polish Holstein–Friesian breed (Table S2).

**Table 1:** Genotype and allele frequencies of the g.−818_−814AGCCGIns/Del, g.−212C<A and g.7C<T polymorphisms of the bovine *MEF2D* gene in six breeds of cattle

	g.−818_814AGCCGIns/Del					g.−211C<A					g.7C<T					
Breed	Genotype			Allele		Genotype			Allele		Genotype			Allele		χ^2^ (*P* value)
	ins/ins	ins/del	del/del	AGCCG	−	CC	CA	AA	C	A	CC	CT	TT	C	T	
HO (203)^a^	0.586 (119)	0.374 (76)	0.040 (8)	0.773	0.227	0.586 (119)	0.374 (76)	0.04 (8)	0.773	0.227	0.586 (119)	0.374 (76)	0.04 (8)	0.773	0.227	0.94 (0.331)
RP (47)	0.553 (26)	0.447 (21)	0.000 (0)	0.777	0.223	0.553 (26)	0.447 (21)	0.000 (0)	0.777	0.223	0.553 (26)	0.447 (21)	0.000 (0)	0.777	0.223	3.83 (0.048)*
HH (34)	0.471 (16)	0.500 (17)	0.029 (1)	0.721	0.279	0.471 (16)	0.500 (17)	0.029 (1)	0.721	0.279	0.471 (16)	0.500 (17)	0.029 (1)	0.721	0.279	1.99 (0.158)
CH (35)	0.629 (22)	0.257 (9)	0.114 (4)	0.757	0.243	0.629 (22)	0.257 (9)	0.114 (4)	0.757	0.243	0.629 (22)	0.257 (9)	0.114 (4)	0.757	0.243	3.17 (0.0751)
SM (29)	0.481 (14)	0.519 (15)	0.000 (0)	0.741	0.259	0.481 (14)	0.519 (15)	0.000 (0)	0.741	0.259	0.481 (14)	0.519 (15)	0.000 (0)	0.741	0.259	3.53 (0.060)
LM (27)	0.407 (11)	0.556 (15)	0.037 (1)	0.685	0.315	0.407 (11)	0.556 (15)	0.037 (1)	0.685	0.315	0.407 (11)	0.556 (15)	0.037 (1)	0.685	0.315	2.24 (0.134)

^a^In bracket, number of animals; Genotypes: ins/ins = AGCCG/AGCCG; ins/del = AGCCG/−; del/del = −/−

* *P* < 0.05

**Table 2:** Combined genotype frequencies of the g.−818_814AGCCGIns/Del, g.−211C<A and g.7C<T polymorphisms of the bovine *MEF2D* gene in six breeds of cattle

Combined genotype	Breed					
	Holstein–Friesian (203)^a^	Polish Red (47)	Hereford (34)	Charolaise (35)	Simmental (29)	Limousine (27)
Ins-C-C/Ins-C-C	0.586 (119)	0.553 (26)	0.471 (16)	0.629 (22)	0.481 (14)	0.407 (11)
Del-A-T/Del-A-T	0.040 (8)	0.000 (0)	0.029 (1)	0.114 (4)	0.000 (0)	0.037 (1)
Ins-C-C/Del-A-T	0.374 (76)	0.447 (21)	0.500 (17)	0.257 (9)	0.519 (15)	0.556 (15)

*n* number of animals

^a^In bracket, number of animals

## Discussion

Members of the *MEF2* family of transcription factors are up-regulated during skeletal muscle differentiation and cooperate with the *MyoD* family of myogenic basic helix-loop-helix (bHLH) transcription factors to control the expression of muscle-specific genes [1, 2]. Recently, several studies have clearly shown that *MEF2* factors are involved in the postnatal regulation of skeletal muscle development, growth and homeostasis [8, 14]. After birth, *MEF2A*, -*B* and -*D* transcripts are expressed ubiquitously, while *MEF2C* transcripts are restricted to skeletal muscle, brain, and spleen. Musaro et al. [15] showed that increases of the *MEF2C* expression in adults and senile mice were associated with increasing expression of the slow myosin isoform, indicating the possible role of *MEF2C* in the induction of the myogenic pattern specific for type I fibers in mature muscles [6, 7, 16]. It is also known that MEF2 proteins act as major transducers of Ca^2+^ signalling events, which play a vital role in the hypertrophic growth and remodelling of adult skeletal muscle in response to mechanical load [17], which might imply that postnatal skeletal muscle growth depends more on Ca^2+^ signalling and MEF2 proteins than on the myogenic bHLH factors [14].

During the last few decades, advances in molecular genetics have led to the identification of genes which influence meat production and quality in farm animals. Many important traits such as carcass and meat quality are controlled by multiple genes and complex gene interactions. The study of candidate genes can be useful to determine whether specific genes are related to the economic traits. It is known that, gene sequences and variations in the regulatory and structural regions are the entry points to study gene expression and function.

In the current study, the promoter region and 5′UTR of the bovine *MEF2D* gene were resequenced and three novel polymorphisms were identified in *Bos taurus* cattle. The distribution of polymorphisms showed diversity among the different cattle breeds, thus indicating a higher frequency of the Ins/Ins, CC and CC genotypes for each locus in the HO, RP and CH breeds as compared to their lower frequency in the HH, SM and LM breeds. This diversity may be due to the difference in breed productivity and breeding purpose. The bovine *MEF2D* gene consists of twelve exons encoding a 507-amino-acid protein, whose amino acid sequence is highly homologous with the MEF2D proteins in humans, mice and other mammals [18]. This implied that the *MEF2D* genes were highly conserved in certain mammals and that the bovine *MEF2D* gene might have similar or even the same functions as the *MEF2D* genes of other mammals. Knowledge on *MEF2* genes polymorphism is limited, and little is known about its effect on gene expression levels, growth and muscle development in farm animals. Several SNPs, which have been associated with hypertrophic cardiomyopathy [19] and coronary artery disease [20, 21], were identified in the human *MEF2A* gene. In our previous study we found two substitutions and two insertion/deletion polymorphisms in the bovine *MEF2C* promoter region, as well as four SNPs in intron 1 [22]. So far, the potential effect of polymorphisms in the regulatory region of the *MEF2D* gene on its expression in the muscle of cattle has not been reported. In the current study we observed that *MEF2D* promoter variants are associated with *MEF2D* mRNA levels and protein abundance in the *longissimus dorsi* muscle of 12-month-old Polish Holstein–Friesian bulls. We have shown that allele-dependent differences in the *MEF2D* gene expression level exist in favour of the Ins-C-C/Ins C-C combined genotype over the Del-A-T/Del-A-T genotype. These results suggested that the g.−818_−814AGCCG and g.−212C<A polymorphisms, which in silico disrupt the binding sites for the *RAF*, *Sp1*, *AP2* and *AP*-*alpha* transcription factors, might be involved in the *cis*-regulation of *MEF2D* transcription in the skeletal muscles, and that gene expression might also depend on the interplay between these transcription factors. It is known that *Sp1* and *AP2* transcription factors play an essential role in the regulation of gene expression during embryogenesis [23, 24]; but also, as shown by Adamowicz et al. [25], the decreased *Sp1* binding capacity affects *LEP* expression in the adipose tissue of adult cattle. Similar effects have been previously reported for other bovine genes, such as *STAT5A* or *IGF*-*1*, where mutations localized in the promoter region changed the affinity of transcription factors to the promoter sequence and acted as *cis*-regulators on the expression of the target gene [26, 27]. Recently, we found that *MEF2A* promoter variants are associated with different *MEF2A* mRNA levels in the muscle of Polish Holstein–Friesian bulls [28]. However, the g.7C<T transition in the 5′UTR of the *MEF2D* mRNA might have an effect on the efficiency of *MEF2D* expression by regulation of mRNA stability or translation efficiency [29]. In addition, these variations might be in linkage disequilibrium with another SNP not screened in the study, e.g. in the 3′UTR of the *MEF2D* gene, which may affect the translation process and/or protein folding, thereby resulting in an altered function of the protein [30]. Only two studies have been performed on the effect of *MEF2* gene polymorphisms on carcass quality traits in domestic animals. Recently, Zhou et al. [31 **]** described the SNPs in the 5′UTR, exon 4 and intron 7 of the chicken *MEF2A* gene which have been associated with carcass traits in chickens. Furthermore, Chen et al. [32] reported that three SNPs in exon 11 of the *MEF2A* gene affect early growth and body weight in Chinese cattle breeds. Our association analysis showed a statistically insignificant effect of the *MEF2D* genotypes on the carcass quality traits of Polish Holstein–Friesian bulls. Nevertheless, it should be noted that interpretation of the results is limited by the low frequency of the Del-A-T/Del-A-T combined genotype in the examined population of cattle. Therefore, further studies should be conducted on a larger population of cattle to confirm the polymorphisms’ usefulness for the marker-assisted selection of cattle.

## Electronic supplementary material

Below is the link to the electronic supplementary material.

Supplementary material 1 (DOC 46 kb)

Supplementary material 2 (DOC 39 kb)

Supplementary material 3 (TIFF 4454 kb)

Supplementary material 4 (TIFF 883 kb)
