# Supplementary material for: Effects of new polymorphisms in the bovine myocyte enhancer factor 2D (MEF2D) gene on the expression rates of the longissimus dorsi muscle
Source: Mol Biol Rep. 2012 Jun 20;39(8):8387–93. doi: 10.1007/s11033-012-1689-6 (PMC3383949; doi:10.1007/s11033-012-1689-6)
Supplement: Supplementary file 1 — Supplementary material 1 (DOC 46 kb) [file 11033_2012_1689_MOESM1_ESM.doc]

**Table S1** Primer pairs for PCR-MSSCP and Real-time PCR analysis of the *MEF2D* gene.

| Locus | Primer sequences | | GenBank/  reference | | Mutation | Region | Position | Length | Tm (°C) |
| --- | --- | --- | --- | --- | --- | --- | --- | --- | --- |
| P1 | F: TTCCACTGAACTTGCCTCCT  R: GAAAGGGGCTGCATGTTATG | | JQ901405 | | *-* | promoter | 1-356 | 356 | 60.0 |
| P2 | F: GTGCCTGTGTTCCAGTTAAT  R: GGACAACTGACCCTGTCAGACT | |  |  | - | promoter | 252-569 | 317 | 59.5 |
| P3 | F: GAGGATTCACCACACCCTGG  R: CGAAGCCCCACTTAAAGAGA | |  |  | AGCCGIns/Del | promoter | 561-936 | 451 | 61.0 |
| P4 | F: CCCAGGTAACCCCCTAGAAA  R: AGGCCTCTAAAGGGATCAGC | |  |  | - | promoter | 889-1204 | 315 | 59.0 |
| P5 | F: CCCCACTTTGTCTTGCTCTG  R: TGCTTCCTTCCTCCTTTCAG | |  |  | C<A | promoter | 1218-1791 | 379 | 57.0 |
| P6 | F: GCCGAGGCGGCTCGGGCGG  R: GCAGGCGCATTATTTTTCTT | |  |  | C<T | 5’UTR | 1561-1791 | 373 | 60.0 |
| Quantitative real-time PCR analysis | | | | | | | | | |
| *MEF2D* | F: CTGCAGACCATGAACTGAGC  R: ACTTGAGCAGCACCTTGTCC | HQ615718 | | - | | mRNA | 208-530 | 322 | 60.0-61.0 |
| *SF3AI* | F: GCGGGAGGAAGAAGTAGGAG  R: TCAGCAAGAGGGACACAAA | XM_878187.1 | | - | | mRNA |  | 122 | 61.0 |
| *TBP* | F: ACAACAGCCTCCCACCCTAT  R: GTGGAGTCAGTCCTGTGCCG | NM_001075742 | | - | | mRNA | 389-499 | 110 | 60.0 |
| *EEF1A2* | F: GCAGCCATTGTGGAGATG  R: ACTTGCCCGCCTTCTGTG | BC_108110.1 | | - | | mRNA |  | 205 | 60.0 |
