# Supplementary material for: Effects of new polymorphisms in the bovine myocyte enhancer factor 2D (MEF2D) gene on the expression rates of the longissimus dorsi muscle
Source: Mol Biol Rep. 2012 Jun 20;39(8):8387–93. doi: 10.1007/s11033-012-1689-6 (PMC3383949; doi:10.1007/s11033-012-1689-6)
Supplement: Supplementary file 2 — Supplementary material 2 (DOC 39 kb) [file 11033_2012_1689_MOESM2_ESM.doc]

Table S2 Least squares means and standard error for carcass quality traits of different combined genotypes of the *MEF2D* gene in Polish Holstein Friesian bulls.

| Traits | Genotype | | |
| --- | --- | --- | --- |
|  | Ins-C-C/Ins C-C  (*n* = 119) | Ins-C-C/Del-A-T  (*n* = 76) | Del-A-T/Del-A-T  (*n* = 8) |
|  |  |  |  |
| WLVC (kg) | 40.5 ± 0.4 | 39.9 ± 1.1 | 39.7 ± 0.2 |
| WFVC (kg) | 5.6 ± 0.9 | 6.3 ± 0.2 | 6.3 ± 0.1 |
| PLVC (%) | 70.2 ± 2.3 | 68.1 ± 0.8 | 67.0 ± 0.6 |
| PFVC (%) | 9.9 ± 4.1 | 10.7 ± 1.2 | 12.7 ± 0.9 |

*n* ‒ number of animals; WLVC (kg) – weight of lean in valuable cuts (kg); WFVC ‒ weight of fat in valuable cuts; PLVC (%) – percentage of lean in valuable cuts in carcass side; PFVC (%) ‒ percentage of fat in valuable cuts;
