# Supplementary figures and images for: Effects of new polymorphisms in the bovine myocyte enhancer factor 2D (MEF2D) gene on the expression rates of the longissimus dorsi muscle
Source: Mol Biol Rep. 2012 Jun 20;39(8):8387–93. doi: 10.1007/s11033-012-1689-6 (PMC3383949; doi:10.1007/s11033-012-1689-6)

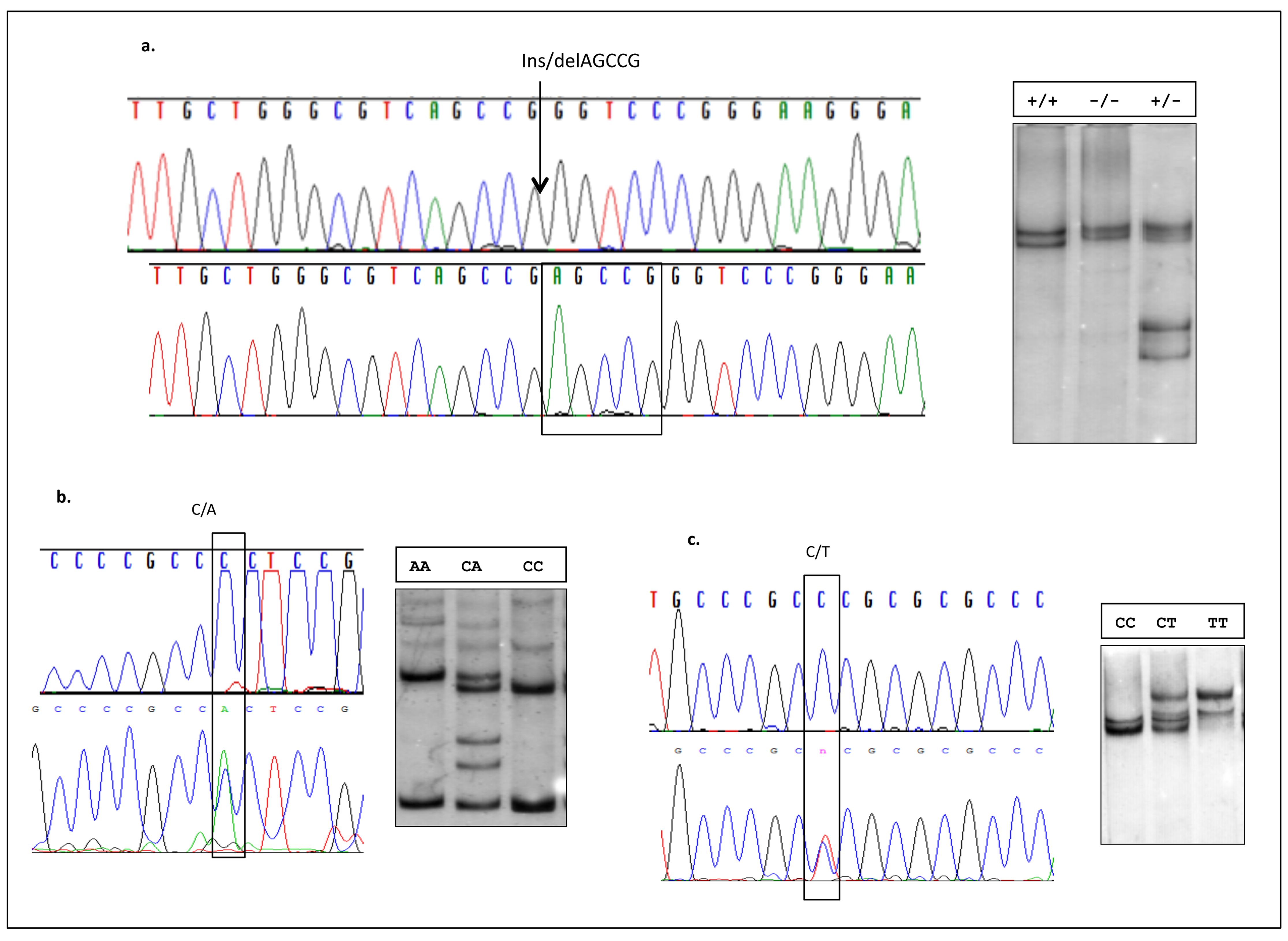

Supplement: Supplementary file 3 — Supplementary material 3 (TIFF 4454 kb) [file 11033_2012_1689_MOESM3_ESM.tif]

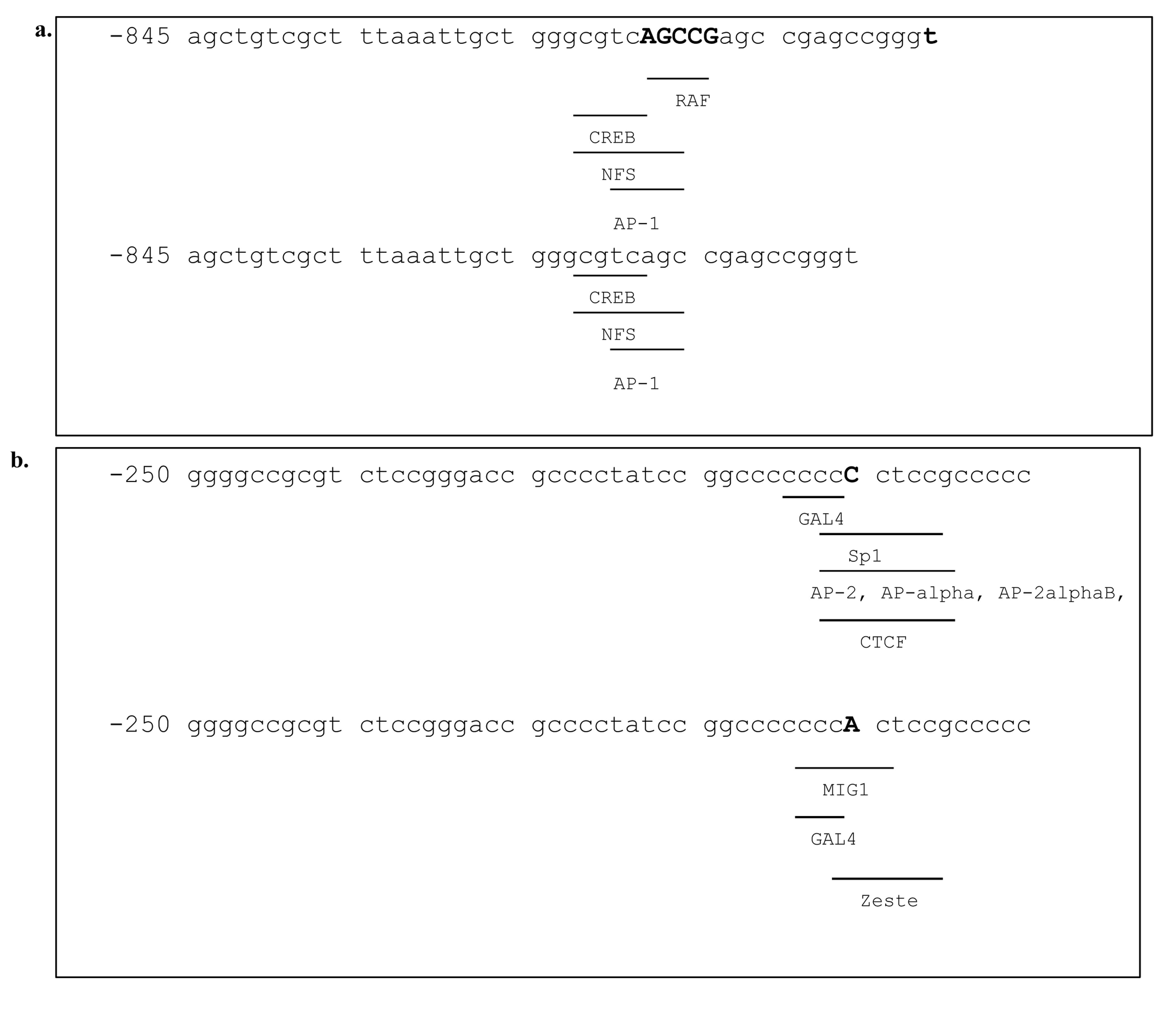

Supplement: Supplementary file 4 — Supplementary material 4 (TIFF 883 kb) [file 11033_2012_1689_MOESM4_ESM.tif]
